# Comparative Analysis of Pelletized and Unpelletized Sunflower Husks Combustion Process in a Batch-Type Reactor

**DOI:** 10.3390/ma14102484

**Published:** 2021-05-11

**Authors:** Tomasz Turzyński, Jacek Kluska, Mateusz Ochnio, Dariusz Kardaś

**Affiliations:** Institute of Fluid Flow Machinery, Fiszera 14, 80-231 Gdansk, Poland; jacek.kluska@imp.gda.pl (J.K.); mochnio@imp.gda.pl (M.O.); dk@imp.gda.pl (D.K.)

**Keywords:** combustion, sunflower husk, hardwood, combustion velocity

## Abstract

This paper describes characteristics of the combustion of sunflower husk (SH), sunflower husk pellets (SHP), and, for comparison, hardwood pellets (HP). The experiments were carried out using a laboratory-scale combustion reactor. A proximate analysis showed that the material may constitute an alternative fuel, with a relatively high heating value (HHV) of 18 MJ/kg. For SHP, both the maximum combustion temperatures (T_MAX_ = 1110 °C) and the kinetic parameters (temperature front velocity v_t_ = 7.9 mm/min, combustion front velocity v_c_ = 8 mm/min, mass loss rate v_m_ = 14.7 g/min) of the process were very similar to those obtained for good-quality hardwood pellets (T_MAX_ = 1090 °C, v_t_ = 5.4 mm/min, v_c_ = 5.2 mm/min, v_m_ = 13.2 g/min) and generally very different form SH (T_MAX_ = 840 °C, v_t_ = 20.7 mm/min, v_c_ = 19 mm/min, v_m_ = 13.1 g/min). The analysis of ash from SH and SHP combustion showed that it has good physicochemical properties (ash melting point temperatures >1500 °C) and is safe for the environment. Furthermore, the research showed that the pelletization of SH transformed a difficult fuel into a high-quality substitute for hardwood pellets, giving a similar fuel consumption density (F_out_ = 0.083 kg/s·m^2^ for SHP and 0.077 kg/s·m^2^ for HP) and power output density (P_ρ_ = MW/m^2^ for SHP and 1.5 MW/m^2^ for HP).

## 1. Introduction

Biomass wastes, as compared to fossil fuels, wood, and wheat straw, may be an interesting alternative renewable energy resource. However, these fuels, in terms of exploitation of their potential, present a challenge for conventional technological solutions. The constant increase in the quantity of biomass-derived waste materials has created the need for an industrial sector to further develop industrial thermal waste management technology based on these types of fuel. One of the most promising animal-derived waste energy sources is poultry litter, as described by [1,2]. Furthermore, an experimental investigation of horse manure combustion has been reported by [3]. Other types of waste, such as sewage sludge, have also been the subject of research [4,5,6,7]. Rice husks are another interesting source of biomass waste. The characteristics of pine wood and rice husk combustion, including temperature and emission measurements, were presented by [8]. According to the literature, many kinds of non-wood biomass are potential alternative energy sources. These fuels also come in different forms, including shells, grains, pits, and grasses, which can often lead to various problems such as slagging, deposit formation, decrease in combustion temperature, or fluctuation in the fuel consumption [9]. Acknowledging that one of the most common solutions used for the domestic combustion of fuels is small fixed-grate furnace units [10], the operational problem related to the combustion process on a grate becomes even more significant. For this reason, the conversion of waste biomass fuel into a pelletized form has become the subject of intense research and many scientific publications.

Sunflower husks, especially those subjected to the pelletization process, seem to be a resource that could successfully supplement traditional wood pellets. This work presents and compares the results of the combustion of sunflower husk (SH) and pelletized sunflower husk (SHP). The pelletization process enables increasing the energy density of the fuel, and can therefore generally improve the quality of the product and increase control over the combustion process.

The possibility of pelletization of rice husks and the characteristics of their combustion were reported by [11]. It had been noted previously that the reduction of fuel size by pelletization leads to an increase in the fuel bulk density and provides fuel size reduction before transportation [12]. This aspect was also presented by [13], Ref. [11] reported and characterized the pelletization of rice husk and wheat straw, while [14] presented a characterization of the pelletization process of poplar and pine sawdust. Ref. [15] showed that the operational cost of pelletization of waste fuels such as alfalfa, sawdust, and pig and chicken feed wood waste is in fact highly variable, ranging from 8 to 75 kWh/tonne depending on the material.

Sunflower husks have also been the subject of many studies, concerning various applications. According to the literature, the sunflower is very commonly grown on every continent, with a total production of 47 million tonnes annually [16]. The weight of the sunflower husk amounts to about 40–60% of the produced seeds. For this reason, sunflower husk is a promising source of pellet production, amounting to about 240,000 tonnes per year in Ukraine, while worldwide agriculture generates over 10 million tonnes of sunflower seed [17]. According to [18] sunflower production in Croatia reached 2.77 t/ha and indicated sunflower husk as a potential high-quality biomass fuel.

The possibility of using ash from sunflower husk in the ceramic industry was described by [19], while [20] presented the use of sunflower husk in the iron ore sintering process. In turn, Ref. [21] presented the characteristics of sunflower husk as a filler for epoxy-based composites, with analysis of the mechanical properties. However, there is a lack of studies describing the combustion process of sunflower husk and its potential as a heat source.

The characteristics of sunflower husks and sunflower husk pellets as fuels for co-firing with brown coal in energy boilers were presented by [22]. Moreover, computational analysis, using CHEMKIN-PRO (Ansys, Canonsburg, PA, USA), and an experimental study using thermal analysis (TGA) were used by [23,24] to analyze sunflower husk pellet and pine wood combustion and their co-combustion with oats.

This work presents a characterization of the dynamics of the direct combustion of pure sunflower husk (SH) and pelletized sunflower husk (SHP), including temperature characteristics, averaged maximum temperatures (T_MAX_), and averaged flame temperatures (T_flame_), as well as combustion kinetics, including temperature front velocity (v_t_) and combustion front velocity (v_c_). For this end, an innovative experimental stand was prepared, allowing for simultaneous temperature measurement inside and above the combusting fuel bed, as well as visual observation of the process. It also includes a characterization of the energy balance, including mass loss rate, as well as two original parameters proposed by the authors: fuel consumption density (F_out_) and power output density (P_ρ_). These new intertwined parameters allow for a direct comparison between vastly different fuel types and provide guidance towards designing proper, dedicated combustion devices or grate furnaces.

## 2. Materials and Methods

### 2.1. Materials

Pelletized sunflower husks were made using a flat die pellet-making machine. The nominal size of the pellets was 8 mm in diameter and about 10 mm in length. Pellets were made without the use of any external additives.

Proximate and ultimate analyses of sunflower husk, sunflower husk pellets, and hardwood pellets are presented in Table 1. Analyses were carried out using a S8 TIGER 1 kW-High Performance Wavelength Dispersive XRF spectrometer (Bruker Scientific Instruments, Billerica, MA, USA) and an Organic Elemental Analyzer Flash 2000 CHNS/O (Thermo Scientific, Waltham, MA, USA). The moisture content was determined using a Moisture Analyzer MAX 50 (Radwag, Radom, Poland; max capacity 50 g, readout accuracy 0.0001%), and calorific value was determined using a calorimeter (EkotechLAB, Gdańsk, Poland).

**Figure 1:**
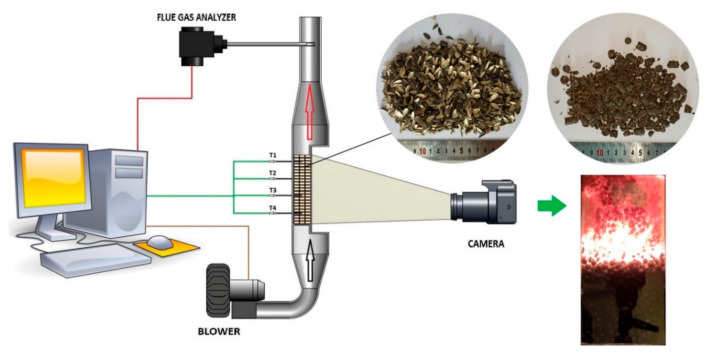
Test stand for the combustion process.

To evaluate the combustion characteristics of these fuels, sunflower husk and sunflower husk pellets (shown in Figure 1) were compared with commercial hardwood pellets (8 mm in diameter and 10–15 mm in length). The measured bulk density of HP was 669 kg/m^3^, while for SHP and SH the respective values were 478 kg/m^3^ and 139 kg/m^3^. All of the materials had a similar HHV value and composition (the analysis of HP shows a slightly higher fixed carbon content at the cost of the volatiles fraction).

### 2.2. Combustion Procedure

Experiments were carried out using a small-scale batch combustion reactor, specially designed for simulating the combustion process on a grate, and equipped with an air supply, a temperature measurement and recording system, and an exhaust gas analyzer (Figure 1). The combustion process in the reactor progressed from the top of the packed bed to the bottom, along a vertical trajectory. The combustion reactor and exhaust tube were insulated with 3 cm insulating rock wool. The laboratory reactor’s combustion chamber had a half- cylinder shape, with a diameter of 80 mm and height of 200 mm. The front, flat wall of the combustion chamber was covered with heat resistant glass, enabling visual recording of the process (camera in Figure 1). Four K-type thermocouples (T1–T4, measurement range −40–1200 °C, class 1, shielding material—AISI 314 (Termoaparatura Wrocław, Poland)) were located horizontally above the grate at equal distances from each other (40 mm) and from the curved wall of the combustion chamber (25 mm). A grate with 24 holes, each of 4 mm, was placed at the bottom of the combustion chamber. Temperature measurements were taken at 30 s intervals. The results obtained for the temperature distribution inside the fuel bed during combustion enabled determination of the temperature front velocity (v_t_), as well as the maximum combustion temperature T_MAX_ and flame temperature T_flame_. In a batch-type combustion reactor, given that the process is stable, the temperature front travels through the fuel bed at a certain constant rate. This rate is called the temperature front velocity (v_t_) and can be calculated using the data provided by the thermocouples (T1–T4). In this study, the researchers focused on the velocity at which the temperature 400 °C “traveled” through the combusting bed. When the distance traveled by the 400 °C front (based on the known distances between thermocouples) is plotted against time, the temperature front velocity v_t_ can be defined as the slope of a linear approximation of these data. For each kind of fuel combusted inside the reactor, the process was repeated three (SHP and HP) or four (SH) times to verify the repeatability of the experiment.

Moreover, the combustion front velocity v_c_ was determined using the methodology previously described by [25]. This original method requires the visual capture of the combustion process inside the reactor at known time intervals (30 s). For this purpose the heat-resistant measurement window was utilized. A camera captures the combustion process in a series of images; then a software tool acquires a color palette from an image not containing any flame or embers, and subtracts that palette from an image of an actual experiment. The result is an image containing only the glow from the combustion process. The location of the lowest pixel visible on the image is the location of the combustion front at that moment in time. The slope of a linear approximation of these locations over time is called the combustion front velocity v_c_. This parameter provides information about whether or not the v_c_ parameter was calculated correctly and combined with temperature profiles can be used to determine the amount of afterburn occurring for each fuel. During each experiment, the weight loss of SH, SHP, or HP was monitored and recorded.

In the experiments, the total mass of the sample inserted into the reactor, according to bulk density, was approximately 500 g for HP, 350 g for SHP, and 100 g for SH. Each experiment was conducted several times (3 or 4) to ensure repeatability. The flow rate of the air supplied for the combustion for each kind of fuel was adjusted so that the amount of carbon monoxide (CO) in the flue gases was minimal and the process occurred in over-stoichiometric conditions (λ > 1).

To ignite the sample, the surface of the bed was covered with a layer of highly volatile cellulose cubes. After air was supplied from the bottom of the reactor, an open flame was applied to the cubes. Upon obtaining a stable combustion process of the fuel bed, signified by a constant mass loss rate, the experimental investigation was conducted.

## 3. Results and Discussion

### 3.1. Temperature Characteristics

**Figure 2:**
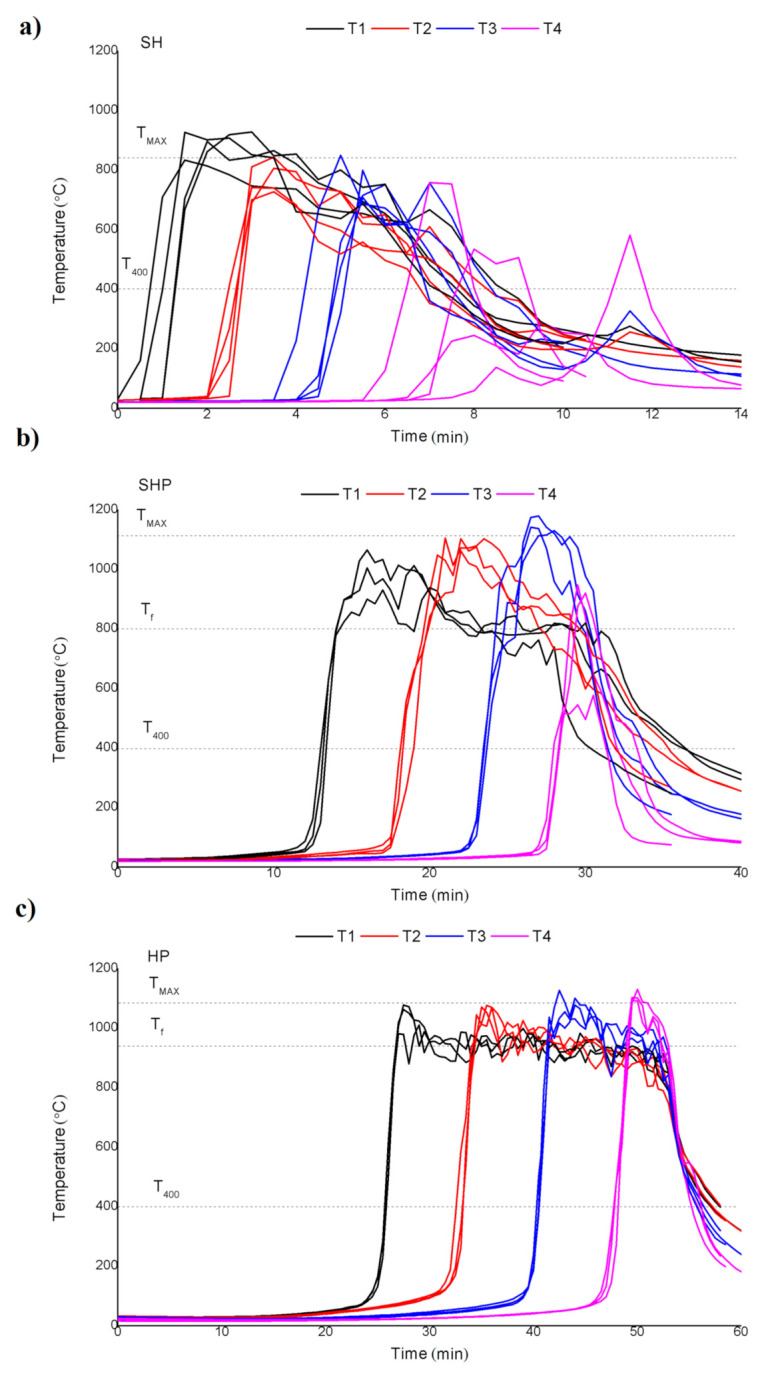
Temperature characteristics for combustion of (**a**) sunflower husk, (**b**) sunflower husk pellets, and (**c**) hardwood pellets.

The following paragraphs present a comparison of the combustion processes of sunflower husk, sunflower husk pellets, and wood pellets. Analysis of the experimental data (Figure 2) places the averaged maximum temperatures (T_MAX_) during the combustion process at 840 °C for sunflower husk, 1110 °C for sunflower husk pellets, and 1090 °C for wood pellets. Similarly, the averaged flame temperatures (T_flame_) during the combustion process were approximately 800 °C for sunflower husk pellets and 940 °C for wood pellets. However, the sunflower husk combustion process did not produce a stable flame temperature, suggesting that the process itself was not stable. Closer inspection of the temperature profiles seemed to corroborate this observation. The temperature spikes for each batch were not evenly distributed during the experiment, which implies that the combustion process occurred at different, randomized rates for each experiment. This was probably because of the relatively low bulk density of the fuel itself. During combustion, individual husks became light enough for the air flow to cause mixing of the bed in the reaction zone, thus introducing irregularities in the bed and affecting the speed of the process. In some instances, light, unburnt particles were even carried out of the reactor by the air flow. On the other hand, for both SHP and HP the combustion process progressed at a constant rate, and the result was repeatable for each batch. Nevertheless, the temperature spikes corresponding to SHP did not align as well as the temperature spikes for HP. In addition, the difference between maximum temperature and flame temperature for SHP was higher than for HP, which suggests that using SHP as a primary fuel may result in lower thermal parameters for the combustion device and heat exchanger. Notably, a lower operational load of the boiler using SHP was also independently corroborated by [26].

### 3.2. Combustion Kinetics

#### 3.2.1. Temperature Front Velocity and Combustion Front Velocity

In a bed composed of HP the temperature front traveled the slowest, at a rate of 5.4 mm/min. The value was slightly higher for SHP (7.9 mm/min) and highest for SH (20.7 mm/min). Furthermore, the values of v_t_ calculated for each batch differed the most for SH, ranging from 17.3 to 22.9 mm/min. A bed composed of SH burned out the fastest, but in a random and highly unstable manner; therefore the v_t_ value obtained from the experiment was also highly inaccurate, although it is somewhat informative.

The combustion front velocities for HP and SHP were 5.2 mm/min and 8.0 mm/min, respectively; corresponding with the temperature front velocities v_t_ obtained from the temperature measurements (5.7 mm/min and 7.9 mm/min). The values of v_c_ for SH, however, again varied widely between batches, ranging from 15 mm/min to 22.4 mm/min, with an average of 19 mm/min. The results showed that the combustion front velocity (v_c_) obtained using the described methodology corresponded to the measured temperature front velocity (v_t_).

#### 3.2.2. Mass Loss

**Figure 3:**
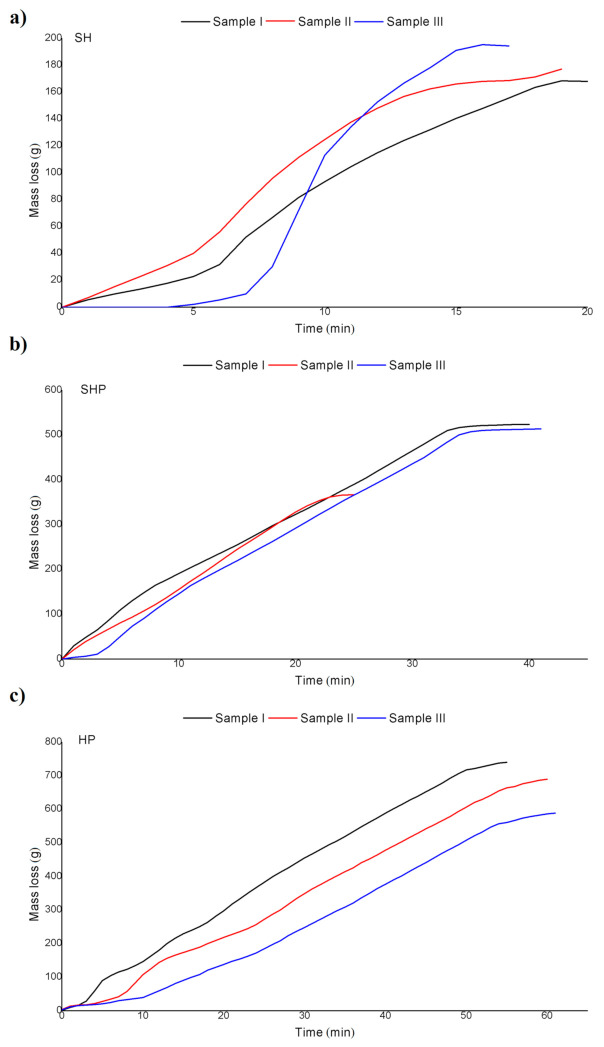
Mass loss rate during combustion of (**a**) sunflower husk, (**b**) sunflower husk pellets, and (**c**) hardwood pellets.

The mass loss of the sample during the combustion process was recorded using a laboratory scale (Radwag APP 30/2C/1, Radom, Poland (Radwag, maximum load 30 kg, measurement accuracy 0.1 g, linearity ± 0.3 g)). Figure 3 shows how the mass of a sample changed over time during the experiment. As mentioned above, each batch was ignited using a layer of flammable material; therefore, the character of mass loss at the early stages of the experiment mainly represented the combustion process of the ignition material. After the ignition, the process of mass loss became stable (linear). Similarly to v_t_ and v_c_, the mass loss rate (v_m_) was calculated as the slope of a linear approximation of the mass loss data over time, taken for a stable combustion process. Both HP and SHP samples produced fairly comparable, repeatable results, while SH samples produced highly diverse results: the ranges were 12.9–13.3 g/min for HP, 12.8–15.8 g/min for SHP, and 8.5–15.7 g/min for SH. However, the averaged v_m_ value appeared to be fairly similar for all fuel types: 13.2 g/min for HP, 14.7 g/min for SHP, and 13.1 g/min for SH.

### 3.3. Energy Balance

Results of calorific analysis of the samples are presented in Table 2. The HHV values of SHP and HP are very similar, while for pure SH the value is slightly lower. On the other hand, the bulk densities of the fuel types are very different, ranging from 139 kg/m^3^ for SH, through 478 kg/m^3^ for SHP, to 669 kg/m^3^ for HP. Other studies on sunflower husk pellets have shown various SHP bulk densities ([27], 838 kg/m^3^, Ref. [28], 540 kg/m^3^, Ref. [26], 529 kg/m^3^), which the pelletization process can significantly alter, depending on the size of the die and the compression rate.

To characterize the energy output of the reactor, two original parameters, the fuel consumption density F_out_ and power output density P_ρ_, were defined as:

(1) $$F_{\mathrm{out}}=\frac{v_{m}}{A_{g}}\;$$

(2) $$P_{\rho }={\mathrm{HHV}}_{\mathrm{avr}}\;\cdot \;F_{\mathrm{out}}\;$$

where A_g_ (m^2^) is the surface area of the grate.

The fuel consumption density F_out_ describes how much mass of the fuel a square meter of a grate combusts every second (Equation (1)), while the power output density P_ρ_ shows how much power is released from that same square meter (Equation (2)). Considering that the v_m_ value is highest for SHP, this means that SHP also has the highest fuel consumption density (F_out_ = 0.083 kg/s·m^2^) and power output density (P_ρ_ = 1.59 MW/m^2^). The results show that the pelletization process of SH transformed a difficult fuel into a high-quality substitute for hardwood pellets, giving a similar fuel consumption density (F_out_) and power output density (P_ρ_).

### 3.4. Analysis of Bottom Ash

Table 3 presents the inorganic compounds identified in the ash from the combustion processes of sunflower husk and sunflower husk pellets. Characteristics of ash composition were carried out using an S8 TIGER 1 kW-High Performance Wavelength Dispersive XRF spectrometer (Bruker Scientific Instruments, Billerica, MA, USA). Analysis of the ash from SH and SHP combustion showed that it has good physicochemical properties and is safe for the environment. Moreover, ash samples from SH and SHP had similar contents of inorganic compounds. These included potassium oxide (33% for the SH ash sample and 35% for SHP), calcium oxide (25% for SH and 37% for SHP), phosphorus pentoxide (14% for SH and 5% for SHP), and magnesium oxide (13% for SH and 11% for SHP). According to the literature, differences in the composition of ash from sunflower husk and sunflower husk pellets may be caused by additives used during the pelleting process [22]. The obtained results of ash composition were also similar to results presented by [28] and showed that ash from biofuels may be used as a soil amendment. In this case it is important to analyze the pollutants content, which is related to environmental protection. In turn, Ref. [19] showed the possibility of utilizing a similar ash from sunflower husks to develop new glass/crystal materials.

In addition, the characteristic temperatures of the bottom ash were determined (according to the methodology of PN-G-04535:1982). The results are given in Table 4. The obtained characteristic temperatures were further compared with those of other biomass fuels for which the DIN 51730 was applied [29,30]. A low plasticization temperature of the ash can lead to sticking to the grate or slagging. Moreover, low spherical and hemispherical temperatures can lead to the sticking of fly ash to the boiler heat exchangers. However, for SHP, both the melting temperature and the spherical and hemispherical temperatures were quite high, comparable even to the values for pine wood or willow wood. The results show that ash from sunflower husk and from sunflower husk pellets is characterized by high melting temperatures, comparable to those of high-quality fuels. Interestingly, the initial deformation temperature was lower by 130 °C for SH than for SHP, which consists basically of the same material. The pelletization of the sunflower husk led to an increase in the initial deformation temperature, from 970 °C for SH to 1100 °C for SHP. Additionally, the acquired temperatures regarding SHP ash correspond with an independent study by [28].

## 4. Conclusions

This paper has provided a comparative characterization of the batch combustion, on a grate, of sunflower husk, sunflower husk pellets, and hardwood pellets. The experimental research used an innovative laboratory-scale reactor and incorporated several original parameters proposed by the authors: temperature front velocity v_t_, combustion front velocity v_c_, fuel consumption density F_out_, and power output density P_ρ_. These parameters provide vital data regarding fuel behavior on a grate and can be used to design an optimal combustion method for a particular fuel.

The analysis shows that sunflower husk may constitute a good-quality alternative fuel, with a relatively high heating value. It was shown that for sunflower husk pellets, the maximum combustion temperatures and the kinetic parameters of the combustion process are very similar to those obtained for good-quality hardwood pellets. On the other hand, the combustion of unpelletized sunflower husk produced a lower average maximum temperature and average flame temperature, and it was found that the combustion process was not stable, due to the relatively low bulk density of the fuel and irregularities in the bed, which affected the speed of the process, and the lifting and carrying of light particles by the air flow. It was concluded that the pelletization of sunflower husk, despite the initial costs, transformed a difficult fuel into a high-quality substitute for hardwood pellets; giving similar fuel consumption density and power output density. Furthermore, the pelletization process approximately triples the bulk density of the fuel, which is very important for storage and logistics. The analysis of ash from SH and SHP combustion showed that it has good physicochemical properties and is safe for the environment. Ash from sunflower husk and from sunflower husk pellets is characterized by high melting temperatures, comparable to the value for pine wood, and higher than for wheat straw or rice straw.

Sunflower husk in a pelletized form presents itself as a promising fuel. This article however focuses on a laboratory scale combustion process with fairly restrictive assumptions. Further research, due to the importance of legislation and environmental protection, should include a standard fixed grate pellet burner and a boiler unit. Such an experimental stand would provide more information regarding particle emission, flue gas composition, ash production, and fouling of the grate/heat exchanger. It would be possible to determine the impact of this fuel on the overall performance of the unit compared with standard hardwood pellets. Moreover, some insight into the pellet production process would also be desirable, as the quality of the fuel, especially its bulk density and rigidity, could surely be improved.

## Figures and Tables

**Table 1:** Proximate and ultimate analysis of sunflower husk and hardwood pellets.

Parameter	Sunflower Husk	Sunflower Husk Pellets	Hardwood Pellets
HHV (MJ/kg)	18.11	19.18	19.60
Moisture (wt%), as delivered	9.61	10.71	6.1
Proximate (wt.%_db_) ^a^			
Volatiles	82.7	83.59	76.3
Fixed carbon	16.1	14.51	21.4
Ash	1.2	1.9	2.3
Ultimate (wt.%_db_) ^a^			
C	46.21	43.38	48.50
H	6.06	6.62	5.30
O	46.58	48.81	45.56
N	0.88	1.19	0.40

^a^ db = oven-dry basis.

**Table 2:** Parameters of the operation of the batch combustion reactor using sunflower husk, sunflower husk pellets, and hardwood pellets.

Parameter	Sunflower Husk	Sunflower Husk Pellets	Wood Pellets
HHV_avr_ (MJ/kg)	18.11	19.18	19.60
v_c_ (mm/min)	19.0	8.0	5.2
v_m_ (g/min)	13.2	14.7	13.1
ρ_bulk_ (kg/m^3^)	139	478	669
T_MAX_ (°C)	840	1110	1090
T_flame_ (°C)	-	800	940
F_out_ (kg/s·m^2^)	0.072	0.083	0.077
P_ρ_ (MW/m^2^)	1.30	1.59	1.50

**Table 3:** Inorganic compounds identified in ash from the combustion of sunflower husk and sunflower husk pellets.

Compound	Sunflower Husk	Sunflower Husk Pellets
K_2_O	33.97	35.14
CaO	25.50	37.40
P_2_O_5_	14.58	5.38
MgO	13.92	11.73
SO_3_	5.14	5.66
SiO_2_	3.94	1.92
Fe_2_O_3_	1.49	0.99
Al_2_O_3_	0.77	0.21
Cl	0.21	0.85
MnO	0.20	0.20
SrO	0.1	0.09
CuO	0.08	0.07
ZnO	0.08	0.11

**Table 4:** Properties of ash from sunflower husk and sunflower husk pellets and from various types of biomass [29,30].

Ash	Initial Deformation Temperature (°C)	Spherical Temperature (°C)	Hemispherical Temperature (°C)	Fluid Temperature (°C)
Sunflower husk	970	>1500	>1500	>1500
Sunflower husk pellets	1100	1490	>1500	>1500
Pine wood	1190	1200	1220	1280
Wheat straw	850	1040	1120	1320
Rice straw	860	980	1100	1220
Willow wood	1380	1540	1550	1560

## Data Availability

The data presented in this study are available upon request from the corresponding author.

## Nomenclature

SH	Sunflower husk
SHP	Sunflower husk pellets
HP	Hardwood pellets
HHV	Higher heating value of the fuel (MJ/kg)
T_flame_	Averaged flame temperature (°C)
T_MAX_	Maximum combustion temperature (°C)
v_t_	Temperature front velocity (mm/min)
v_c_	Combustion front velocity (mm/min)
v_m_	Mass loss rate (g/min)
F_out_	Fuel consumption density (kg/s·m^2^)
P_ρ_	Power output density (MW/m^2^)
T1–T4	Thermocouple 1–4
A_g_	Surface area of the grate (m^2^)
ρbulk	Bulk density of the fuel bed (kg/m^3^)
